# Identification of deleterious recessive haplotypes and candidate deleterious recessive mutations in Japanese Black cattle

**DOI:** 10.1038/s41598-021-86225-y

**Published:** 2021-03-23

**Authors:** Shinji Sasaki, Toshio Watanabe, Takayuki Ibi, Kiyotoshi Hasegawa, Yoichi Sakamoto, Shunsuke Moriwaki, Kazuhito Kurogi, Atsushi Ogino, Takanori Yasumori, Hiroyuki Wakaguri, Eiji Muraki, Youko Miki, Yuichi Yoshida, Yoshinobu Inoue, Ichiro Tabuchi, Ken Iwao, Taichi Arishima, Keisuke Kawashima, Manabu Watanabe, Sumio Sugano, Yoshikazu Sugimoto, Yutaka Suzuki

**Affiliations:** 1 Faculty of Agriculture, University of the Ryukyus, 1 Senbaru, Nishihara, Nakagami-gun, Okinawa, 903-0213 Japan; 2 United Graduate School of Agricultural Sciences, Kagoshima University, 1-21-24 Korimoto, Kagoshima, 890-0065 Japan; 3 Maebashi Institute of Animal Science, Livestock Improvement Association of Japan, Inc., Maebashi, 371-0121 Japan; 4 Graduate School of Environmental and Life Science, Okayama University, Tsushima-naka, Okayama, 700-8530 Japan; 5 Shimane Prefecture Livestock Technology Center, Koshi, Izumo, Shimane 693-0031 Japan; 6 Department of Medical Genome Sciences, and Department of Computational Biology, Graduate School of Frontier Sciences, The University of Tokyo, Chiba, 277-8562 Japan; 7 Hida Beef Cattle Research Department, Gifu Prefectural Livestock Research Institute, Makigadou, Kiomi, Takayama, Gifu 506-0101 Japan; 8 Hyogo Prefectural Technology Center for Agriculture, Forest and Fisher, Hokubu Agricultural Technology Institute, Asago, Hyogo 669-5254 Japan; 9 Tottori Prefecture Livestock Research Center, Tohaku-gun, Kotoura-cho 689-2503 Japan; 10 Cattle Breeding Development Institute of Kagoshima Prefecture, Osumi, So, Kagoshima 899-8212 Japan; 11 Shirakawa Institute of Animal Genetics, Japan Livestock Technology Association, Yushima, Bunkyouku, Tokyo 113-0034 Japan

**Keywords:** Genetics, Animal breeding, Genome, Mutation, Sequencing

## Abstract

Intensive use of a few elite sires has increased the risk of the manifestation of deleterious recessive traits in cattle. Substantial genotyping data gathered using single-nucleotide polymorphism (SNP) arrays have identified the haplotypes with homozygous deficiency, which may compromise survival. We developed Japanese Black cattle haplotypes (JBHs) using SNP array data (4843 individuals) and identified deleterious recessive haplotypes using exome sequencing of 517 sires. We identified seven JBHs with homozygous deficiency. JBH_10 and JBH_17 were associated with the resuming of estrus after artificial insemination, indicating that these haplotypes carried deleterious mutations affecting embryonic survival. The exome data of 517 Japanese Black sires revealed that AC_000165.1:g.85341291C>G of *IARS* in JBH_8_2, AC_000174.1:g.74743512G>T of *CDC45* in JBH_17, and a copy variation region (CNVR_27) of *CLDN16* in JBH_1_1 and JBH_1_2 were the candidate mutations. A novel variant AC_000174.1:g.74743512G>T of *CDC45* in JBH_17 was located in a splicing donor site at a distance of 5 bp, affecting pre-mRNA splicing. Mating between heterozygotes of JBH_17 indicated that homozygotes carrying the risk allele died around the blastocyst stage. Analysis of frequency of the *CDC45* risk allele revealed that its carriers were widespread throughout the tested Japanese Black cattle population. Our approach can effectively manage the inheritance of recessive risk alleles in a breeding population.

## Introduction

Japanese Black cattle is a beef breed, which are very popular for the high degree of marbling of their meat due to intramuscular fat deposition^1^. In Japan, stringent selection for marbling in a closed breeding system^2^ using artificial insemination (AI) has resulted in intense inbreeding, few founder animals, and declined effective population sizes among Japanese Black cattle^3^. The structure of inbred populations makes the individuals susceptible to recessive inherited disorders. Eleven recessive disorders have been identified in Japanese Black cattle thus far^4–15^ (see Supplementary Table S1). As elite sires carry these recessive alleles, their frequency may gradually increase, eventually increasing the frequency of homozygous deleterious phenotypes, which in turn will compromise the welfare of animals and cause severe economic losses. Thus, the identification of causative mutations and the development of genetic diagnostic tests are essential for identifying carriers so they can be excluded from breeding programs, preventing risky matings.

Recently, substantial genotyping data using SNP arrays have been gathered in genome-wide association studies^16^ and genomic selection^17^ in cattle, which have allowed for the identification of haplotypes with homozygous deficiency^18^. Regions with homozygous haplotype deficiency in healthy adults may harbor recessive deleterious mutations, which may compromise pre-, peri-, and postnatal survival until adulthood. Using SNP arrays and genomic studies, regions with homozygous haplotype deficiency have been identified in several cattle breeds^18–21^, which ultimately helped detect causal mutations of early embryonic loss^19,21–24^, as well as death or growth retardation of calves^20,21,25,26^ in these regions. However, regions with homozygous haplotype deficiency remain to be evaluated in Japanese Black cattle.

In the present study, we searched for regions with homozygous haplotype deficiency using SNP array data from 4843 individuals and performed whole exome sequencing and copy number variation (CNV) analysis of 517 sires to identify the recessive causal mutations in Japanese Black cattle. We identified seven regions with homozygous haplotype deficiency and three candidate causal mutations.

## Results and discussion

### Regions with homozygous haplotype deficiency in Japanese Black cattle

**Figure 1:**
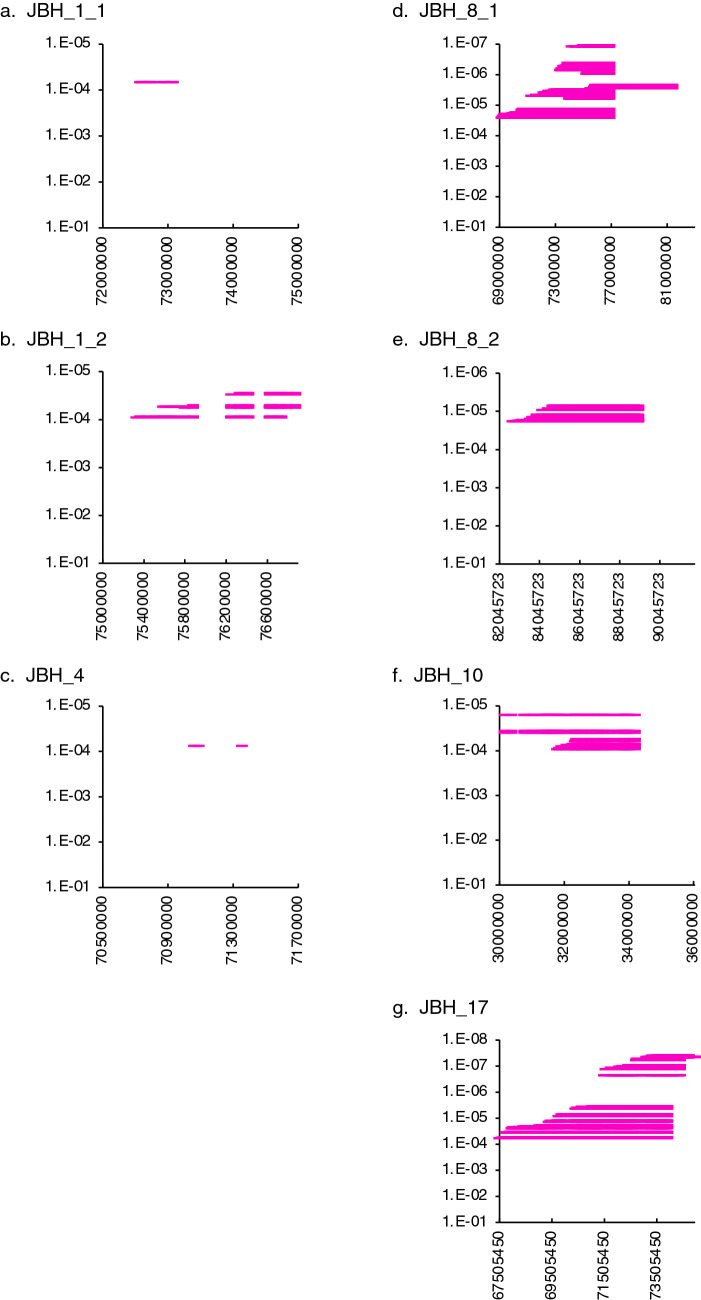
Distribution of the seven haplotypes with homozygous deficiency until an age of 25 months in Japanese Black cattle. (**a**) Japanese Black haplotype (JBH) _1_1. (**b**) JBH_1_2. (**c**) JBH_4. (**d**) JBH_8_1. (**e**) JBH_8_2. (**f**) JBH_10. (**g**) JBH_17. The overlapping haplotypes with a *P* value less than 10^–4^, calculated using the frequencies of homozygotes and carriers with a binomial test, were identified as the risk haplotype regions. Each red horizontal bar represents a haplotype with homozygosity deficiency. Positions on the X-axis are based on the UMD3.1 assembly of the bovine genome.

Haplotypes of 4843 healthy adult Japanese Black cattle (older than 25 months of age) were analyzed by sliding through 2 to 100 SNP windows. The overlapping haplotypes with a *P* value less than 10^–4^, calculated using the frequencies of homozygotes and carriers with a binomial test, were identified as the risk haplotype regions (Fig. 1 and Table 1). We identified seven regions with homozygous haplotype deficiency (Fig. 1, Table 1, and see Supplementary Table S2). The haplotype length was approximately 0.5 to 12.5 Mbp, and the haplotype frequencies ranged from 0.045 to 0.05 (Table 1). We named these haplotype regions using the acronym “JBH” (Japanese Black haplotype) followed by the chromosome number (Table 1). There were no homozygous individuals for JBH_4, JBH_8_1, JBH_8_2, JBH_10, and JBH_17, which was contrary to the expected number of approximately 10 homozygotes per haplotype based on the calculated haplotype frequencies and the sample size of 4843 animals. The observed frequency of homozygotes significantly deviated from the expected frequency, estimated based on the frequency of the risk haplotype (*P* = 5.93 × 10^–5^ to 2.32 × 10^–6^). One homozygote with JBH_1_1 and JBH_1_2 was detected, whereas one homozygote was not significant at the false discovery rate (FDR) threshold (*q* < 0.05). The expected frequency of homozygous haplotypes in 4843 animals was approximately 12 individuals; thus, JBH_1_1 and JBH_1_2 could also harbor the candidate regions (Table 1).

**Table 1:** Regions with homozygous haplotype deficiency and carrier frequencies of potential lethal haplotypes in Japanese Black cattle.

Haplotype_ID^a^	Chr	Start position^b^	End position^b^	Length of haplotype	Number of marker	Risk-haplotype frequency^c^	Number of homozygotes		*P* value^e^
							Expected^d^	Observed	
JBH_1_1	1	72573950	73077982	504,032	10	0.049	11.8	1	9.11^−5^
JBH_1_2	1	75328281	76876908	1,548,627	21	0.049	11.5	1	1.20^−4^
JBH_4	4	70680040	71457286	777,246	6	0.045	9.9	0	4.95^−5^
JBH_8_1	8	68894735	81389800	12,495,065	157	0.046	10.2	0	3.60^−5^
JBH_8_2	8	82687188	88974063	6,286,875	85	0.052	13.0	0	2.32^−6^
JBH_10	10	29787321	34193887	4,406,566	54	0.050	11.9	0	6.41^−6^
JBH_17	17	67505450	74965918	7,460,468	115	0.045	9.7	0	5.93^−5^

^a^Haplotype IDs consist of “JBH” for “Japanese Black haplotype,” followed by chromosome number.

^b^Positions are based on the UMD3.1 assembly of the bovine genome.

^c^Haplotype frequency was calculated in 4,843 individuals.

^d^The expected number of homozygous individuals for a haplotype was calculated using the frequencies of haplotypes and carriers assuming random mating.

^e^*P* value was calculated using the frequencies of homozygotes and carriers with a binomial test.

### Effects of haplotypes on embryonic mortality rate

The regions with homozygous haplotype deficiency may harbor recessive mutations, which may compromise the survival during pre- and postnatal periods. Embryonic mortality was determined in cows that received a second round of AI (2nd AI) at 18–29 days (D), 30–60 D, 61–90 D, 91–140 D, and 141 D–parturition after the first AI (1st AI)^27^. The 2nd AI was performed when estrus resumed after the 1st AI in our AI breeding program. The resuming of estrus after the 1st AI indicated that fertilization or conception derived from the 1st AI had failed. Embryonic mortality was estimated based on 785,993 mating records derived from 79,617 reproductive cows^27^. A significant increase in the number of 2nd AI in mating between a carrier sire and a carrier cow was observed in JBH_10 at 61–60 D and in JBH_17 at 30–60 D after 1st AI relative to other mating types (Table 2). These results indicate that the two haplotypes are associated with embryonic mortality, while the other haplotypes are associated with postnatal mortality until adulthood (less than 25 months of age).

**Table 2:** Effects of haplotypes on embryonic mortality rate.

Haplotype_ID^a^	Mating type	The timing of 2nd AI	Number of non-return	Number of 2nd AI	Embryonic mortality (%)	*P* value
JBH_10	Carrier cow × carrier sire	61–90 days	120	39	25	0.035
	Others		7473	1644	18	
JBH_17	Carrier cow × carrier sire	30–60 days	106	43	29	0.012
	Others		9173	2359	20	

^a^Haplotype IDs consist of “JBH” for “Japanese Black haplotype,” followed by chromosome number.

### Whole exome sequences of 517 Japanese Black sires

**Figure 2:**
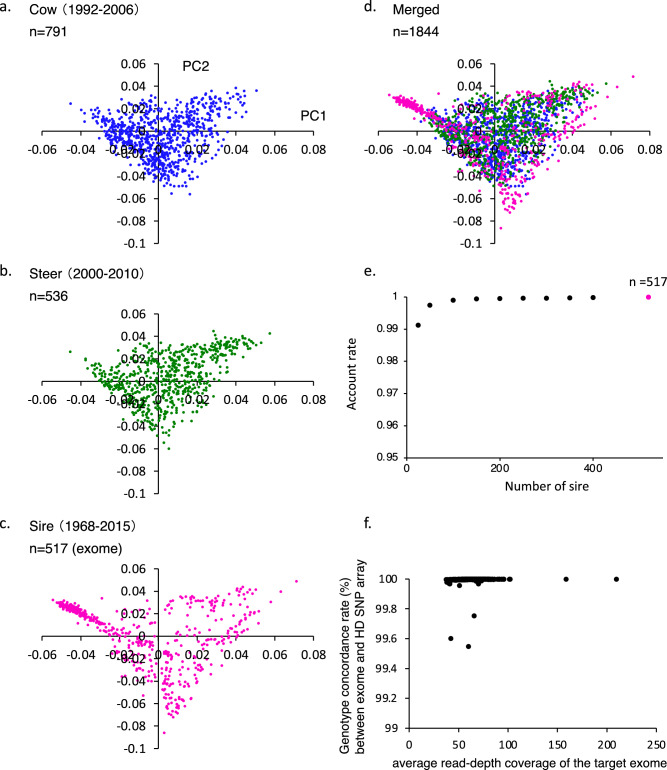
Genetic features and quality of whole exome sequences of 517 Japanese Black sires. (**a**–**d**) Principal component analysis of 1844 Japanese Black cattle, including 791 cows, 536 steers, and 517 sires, was performed using 593,358 autosomal SNPs. The individuals were plotted in a two-dimensional graph, with the first (x-axis: PC1) and the second (y-axis: PC2) principal components. (**d**) Data for 791 cows, 536 steers, 517 exome-sequenced sires were merged. (**e**) Account rate of haplotypes in 25 to 517 sires with the general Japanese Black cattle population. Two consecutive BovineHD SNPs in haplotypes of 517 exome-sequenced sires resulted in an account rate of near 100% with the general Japanese Black cattle population (red plot). The X-axis represents the number of sires, and the Y-axis represents the account rate. (**f**) Quality of exome sequence data of 517 sires. The genotype concordance rate between the exome-derived genotypes and the Bovine High-Density (HD) BeadChip array-derived genotypes was calculated based on 44,772 autosomal SNVs and plotted. The relationships are presented in a two-dimensional graph, with the average read-depth coverage of the target exome (X-axis) and the genotype concordance rate (%) (Y-axis).

To identify deleterious mutations in the risk haplotype, we constructed whole exome sequences of 517 Japanese Black sires, which were born between 1968 and 2015. To evaluate the genetic relationships between the selected 517 sires and general Japanese Black cattle, including cows (1992 to 2006) and steers (2000 to 2010) that were reared at locations across Japan, a principal component analysis (PCA) and haplotype analysis were performed using BovineHD BeadChip genotyped data. The results showed that the 517 sires were clustered with 791 cows and 536 steers in the PCA plot (Fig. 2a–d and see Supplementary Table S3). Additionally, almost all haplotypes harboring the two SNPs derived from the cow and steer populations were shared by the 517 sires (Fig. 2e), indicating that the 517 sires represented the Japanese Black cattle and might be the key sires of the population.

Alignment of filtered exome sequence reads resulted in 61.8-fold read-depth coverage of the target regions in the 517 sires. We evaluated the accuracy of 44,772 SNP calls in the target exomes using genotyped data with the Illumina Bovine High-Density (HD) BeadChip array. The results showed that the average genotype concordance rate was 99.995% (Fig. 2f). Additionally, the exome data confirmed previously reported deleterious mutations in Japanese Black cattle (see Supplementary Table S4), which were detected in all 517 carrier sires, indicating that the exome data could detect deleterious mutations in the risk haplotypes of Japanese Black cattle.

We identified 321,378 single-nucleotide variants (SNVs) and 18,836 indels in the exome data of the 517 sires (Table 3). The variants were annotated using SnpEff^28^ and Ensembl Variant Effect Predictor (VEP)^29^ (Table 3) and compared with data from the 1000 Bull Genomes Projec^30^. We noted that 224,285 SNVs (≈ 70%) in the exome data were identified in the 1000 Bull Genomes Project^30^, while the remaining SNVs (≈30%) were classified as unique in Japanese Black cattle. Next, we used XHMM software^31^, which uses PCA normalization and a hidden Markov model (HMM), to detect CNV based on normalized read-depth data from the exome sequencing data of the 517 sires and CNV regions (CNVRs)^32^ using CNVRuler^33^. Additionally, we used PennCNV^34^ to detect CNVRs using BovineHD BeadChip genotyped data of the 517 sires and an additional 1463 Japanese Black cattle. We detected 5632 CNVRs, of which 1899 were derived from exome data and 4223 from BovineHD BeadChip genotyped data (Table 3), with 490 CNVRs overlapping between the exome and BovineHD BeadChip genotyped data.

**Table 3:** Variant types and annotations from exome sequencing data of 517 sires.

Variants type	Count	Variant annotation	Counts
SNV^a^	321,378	Nonsynonymous	49,986
Indel^b^	18,836	Stop_gained	757
CNVR^c^ (exome + HD)^c^	5632	Frameshift	1362
		Stop_lost	214
		Inframe_insertion	280
		Inframe_deletion	437
		Splice_donor	531
		Splice_acceptor	627
		Synonymous	56,710
		Others	229,310

^a^SNV indicates single-nucleotide variant.

^b^An insertion and a deletion affecting two or more nucleotides.

^c^CNVR indicates copy number variation region.

### Candidate causative mutations of nucleolar protein 6 (*NOL6*) in JBH_8_1 and isoleucyl tRNA synthetase (*IARS*) in JBH_8_2

The numbers of haplotype carriers with JBH_1_1, JBH_1_2, JBH_4, JBH_8_1, JBH_8_2, JBH_10, and JBH_17 were 14, 21, 12, 18, 44, 17, and 22 in the 517 sires, respectively. A previous study on haplotypes reported that the causative variant extended several Mbp outside the identified risk the haplotype region and could be in linkage disequilibrium (LD) with the risk haplotype^19^; thus, we searched for candidate variants both within and beyond (approximately ± 6 Mbp) the risk haplotype region (see Supplementary Table S5). Alignment of sequence reads from the 517 sires against ~ 112.6 Mbp of the total target region identified 17,926 sequence variations (16,739 SNVs and 1187 indels) (see Supplementary Table S5). The causative variants were selected based on the following four criteria: (1) no homozygotes were detected in the 517 sires; (2) the variant allele frequency in the 517 sires was approximately equal to the risk haplotype frequency; (3) the variant was annotated as deleterious by SnpEff, Ensembl VEP, and PolyPhen-2^35^; and (4) the variant was detected in carrier animals. After applying these selection criteria, we detected candidate SNVs in JBH_8_1 and JBH_8_2 (Table 4).

**Table 4:** Candidate variants and CNVRs with homozygous haplotype deficiency in 517 Japanese Black sires and 1463 Japanese Black cattle.

Haplotype_ID^a^	Chr	Variant/CNVR	Genotype		Risk_allele freq	Gene symbol	Annotation	VEP prediction (score)
			Observed	Expected^b^				
JBH_1_1	1	AC_000158.1:g.77469795_77506177del	1840/140/0	1842/135/3	0.035	*CLDN16*	exon 1 ~ 5 deletion	
JBH_1_2	1	AC_000158.1:g.77469795_77506177del	1840/140/0	1842/135/3	0.035	*CLDN16*	exon 1 ~ 5 deletion	
JBH_4	4	Not detected						
JBH_8_1	8	AC_000165.1:g.76542321C>T	435/82/0	438/76/3	0.079	*NOL6*	p.D1009N	Deleterious (0.04)
JBH_8_2	8	AC_000165.1:g.85341291C>G	436/79/0	439/73/3	0.077	*IARS*	p.V79L	Deleterious (0)
JBH_10	10	Not detected						
JBH_17	17	AC_000174.1 g.74743512G>T	473/44/0	474/42/1	0.043	*CDC45*	splice_region_variant	

^a^Haplotype IDs consist of “JBH” for “Japanese Black haplotype,” followed by chromosome number.

^b^The expected number of genotypes was calculated using the frequencies of alleles when assuming random mating.

One SNV in JBH_8_1, AC_000165.1:g.76542321C>T, p.D1009N, was based on ENSBTAT00000004312 and located in exon 24 of *NOL6* (Table 4). *NOL6* is localized in the nucleolus^36^, and its ortholog in yeast is likely involved in the channeling of the nuclear export of aminoacyl tRNA^37^; however, the functions of *NOL6*, including those relevant to embryonic or postnatal mortality, remain unknown. We genotyped 2,720 female Japanese Black cattle with polymerase chain reaction (PCR)–restriction fragment length polymorphism using *BsrG*I. Seven homozygotes with a risk allele frequency (0.073) were found (see Supplementary Table S6), indicating that AC_000165.1:g.76542321C>T is not a causative variant for mortality or incomplete penetrance. Notably, AC_000165.1:g.85826989_85826990dellinsTG, p.H171C in exon 2 of *FGD3* was located on the telomeric side at a distance of 4.4 Mbp from the JBH_8_1 haplotype region (see Supplementary Table S1) and the homozygotes of this variant show skeletal dysplasia and low meat yield^14^, a phenotype that is unfavorable for farmers due to low market value, suggesting that AC_000165.1:g.85826989_85826990dellinsTG may be a candidate causative mutation in the JBH_8_1 haplotype region. However, the allele and the haplotype were not in LD with each other in the 517 sires (*r*^2^ is 0.153) and we detected four homozygotes with the risk-allele in the 517 sires (see Supplementary Table S4); therefore, this variant was not a candidate for causative mutation in the JBH_8_1 haplotype region. Further investigations are warranted to detect the causative mutations in JBH_8_1.

One SNV in JBH_8_2, AC_000165.1:g.85341291C>G, p.V79L, was based on ENSBTAT00000001290 and located in exon 3 of *IARS* (Table 4). This variant has been previously identified as a causative mutation of prenatal mortality^38^ and perinatal weak syndrome in Japanese Black cattle^13^, indicating that this variant was a candidate for causative mutation in JBH_8_2.

### Candidate causative mutations of cell cycle division 45 (*CDC45*) in JBH_17

**Figure 3:**
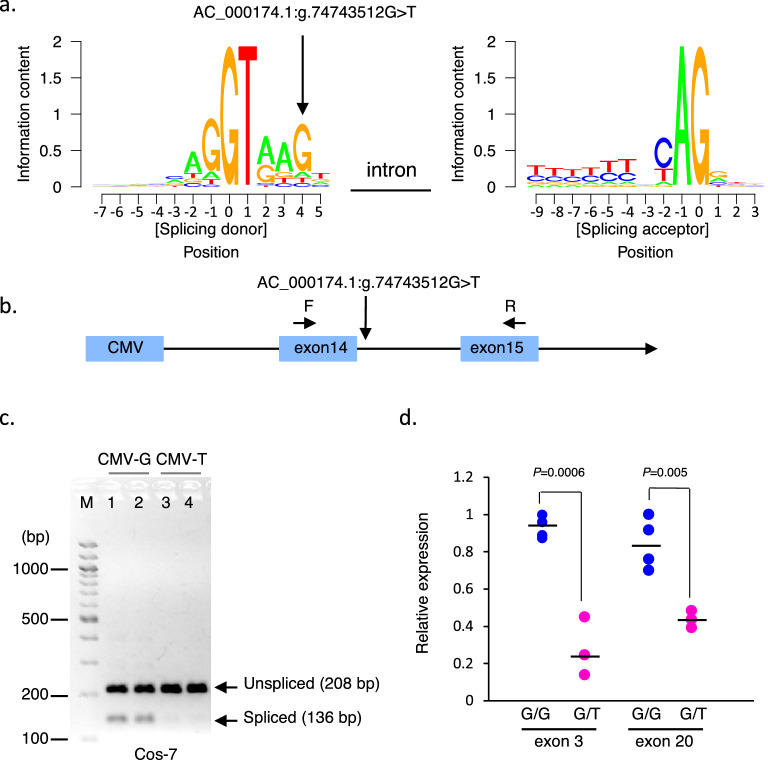
AC_000174.1:g.74743512G>T in JBH_17 Located in the Splicing Donor Site in Intron at a Distance of 5 bp from Exon 14 of *CDC45*. (**a**) Sequences with column heights proportional to the information content of 181,495 introns with GT and 102,705 introns with AG from cattle GTF data derived from the Ensembl database. The vertical arrow indicates the position of AC_000174.1:g.74743512G>T. (**b**) Schematic representation of minigene with exon 14 to exon 15 of *CDC45* including an inter-intron with AC_000174.1:g.74743512G>T and CMV promoter for the splicing assay. The vertical arrow indicates the position of AC_000174.1:g.74743512G>T and the horizontal arrows indicate forward (F) and reverse (R) primers for the splicing assay. (**c**) The minigenes with the reference (G) and risk (T) alleles at AC_000174.1:g.74743512G>T were transfected into Cos-7 cells and amplified using PCR of cDNA with F and R primers shown in (**b**). Expected spliced and unspliced bands were detected at approximately 136 and 208 bp, respectively. (**d**) Relative *CDC45* expression level in the dermal fibroblasts of homozygotes of the reference allele (G) and heterozygotes of the AC_000174.1:g.74743512G>T allele using quantitative PCR with primers specific to exon 3 and exon 20, respectively. *P* values in the graph were calculated using a *t*-test.

No candidate variants in other haplotype regions were found based on our criteria described above; however, we detected one SNV in JBH_17, AC_000174.1:g.74743512G>T, located in the splicing donor site in intron at a distance of 5 bp from exon 14 of *CDC45* (Fig. 3 and see Supplementary Table S7); this variant met all our criteria except that it was not annotated as deleterious. To determine whether the nucleotide in the splicing donor site in intron at a distance of 5 bp from exon 14 of *CDC45* is conserved in exon-intron junctions of cattle, we used SeqLogo^39^ to examine 181,495 exon–intron boundaries with GT and 102,705 intron–exon boundaries with AG based on GTF data derived from the Ensembl database^40^, considering the GT/AG mRNA processing rule^41,42^. The results showed that the G nucleotide is well conserved in the splicing donor site in intron at a distance of 5 bp from exon in cattle (Fig. 3a). To determine whether AC_000174.1:g.74743512G>T affected pre-mRNA splicing of *CDC45*, we constructed a minigene from exon 14 to exon 15 including an inter-intron with AC_000174.1:g.74743512G>T (Fig. 3b) and transfected it into Cos-7 cells. The results confirmed that the level of spliced products with the risk allele (T allele) was significantly lower than that with the reference allele (G allele), indicating that the risk allele affected splicing (Fig. 3c). To compare the relative abundances of transcripts between homozygotes and heterozygotes with the reference alleles, we examined RNA from the primary fibroblasts of Japanese Black cattle using quantitative PCR. The results showed that transcript levels in the heterozygotes were significantly lower than those in the homozygotes with the reference alleles in target regions both upstream and downstream of AC_000174.1:g.74743512G>T (Fig. 3d); therefore, AC_000174.1:g.74743512G>T affects pre-mRNA splicing and mRNA stability of *CDC45*.

By the time a recessive disorder is discovered in a population, the risk allele might already have reached a high frequency in the population. As expected, the frequency of the risk allele was 0.066 in central slaughterhouses (Table 5), indicating that it is already widespread throughout the Japanese Black cattle population in Japan. Although no homozygotes of the risk allele were detected, approximately 7 homozygotes in 1593 animals were expected based on allele frequencies (Table 5). The observed frequency of homozygotes significantly deviated from the expected frequency, which were estimated based on the frequency of the risk allele (chi-square test, *P* = 0.024). Additionally, the carrier sires of the risk allele for *CDC45* were originally reared in a local subpopulation in Japan, implying that mating between heterozygotes with the risk allele is more common in this population than in other populations. The frequency of the risk allele for *CDC45* in cows was 0.084 in the local subpopulation (Table 5). Although no homozygotes of the risk allele were detected, approximately 8.02 homozygotes in 1,137 animals were expected to have the risk allele based on the allele frequencies (Table 5). The observed frequency of homozygotes significantly deviated from the expected frequency, estimated based on the frequency of the risk allele (chi-square test, *P* = 0.002), indicating that the homozygotes of the risk allele for *CDC45* are susceptible to mortality until adulthood.

**Table 5:** Genotypic frequencies of AC_000174.1:g.74743512G>T in *CDC45* in central slaughterhouses and a local subpopulation.

	Central slaughterhouses^a^ (N = 1593)					Local subpopulation (N = 1137)			
	N	Genotype freq	Exp N	Genotype exp freq		N	Genotype freq	Exp N	Genotype exp freq
G/G	1382	0.87	1389	0.87	G/G	946	0.83	954	0.84
G/T	211	0.13	197	0.12	G/T	191	0.17	175	0.15
T/T	0	0.00	7	0.004	T/T	0	0.00	8.02	0.007
	T allele freq = 0.066					T allele freq = 0.084			

^a^Central slaughterhouses in Tokyo Metropolitan Central Wholesale Market, Tokyo, and Nanko Wholesale Market, Osaka.

*CDC45* encodes a component of both pre-initiation (preIC) and helicase complexes required for the initiation of DNA replication and DNA synthesis during the S phase^43–46^. Yoshida et al. reported that *Cdc45* null mouse embryos showed impaired proliferation of the inner cell mass and resulted in embryonic lethality^47^. These reports are consistent with the observed significant increase in the number of 2nd AI in mating between heterozygotes of JBH_17 relative to other mating types at early embryonic stages (Table 2). For further confirmation in cattle, we produced embryos from mating between heterozygotes with the risk alleles. Under a microscope, 34 healthy embryos with homozygous and heterozygous the reference alleles, including 30 hatched embryos and 4 expanded blastocysts were detected, however, no homozygotes for the risk allele were observed (Table 6). The observed frequency of the homozygote significantly deviated from the expected frequency, estimated based on the mating between heterozygotes (chi-square test, *P* = 0.0001). The findings also indicated that the homozygotes of the risk allele in cattle died around the blastocyst stage. Given that it is yet to be determined whether AC_000174.1:g.74743512G>T is directly connected with embryonic mortality, further investigations are necessary to determine the effects of the risk allele on *CDC45* function and pathogenesis in the embryo.

**Table 6:** Genotypes of healthy hatched embryos and expanded blastocytes from mating between heterozygotes with AC_000174.1:g.74743512G>T.

	N	Exp N ^a^
G/G	7	8.5
G/T	27	17.0
T/T	0	8.5

^a^The expected number of embryos were estimated from mating between heterozygotes.

### Candidate causative deletion in Claudin-16 (*CLDN16*) in JBH_1_1 and JBH_1_2

**Figure 4:**
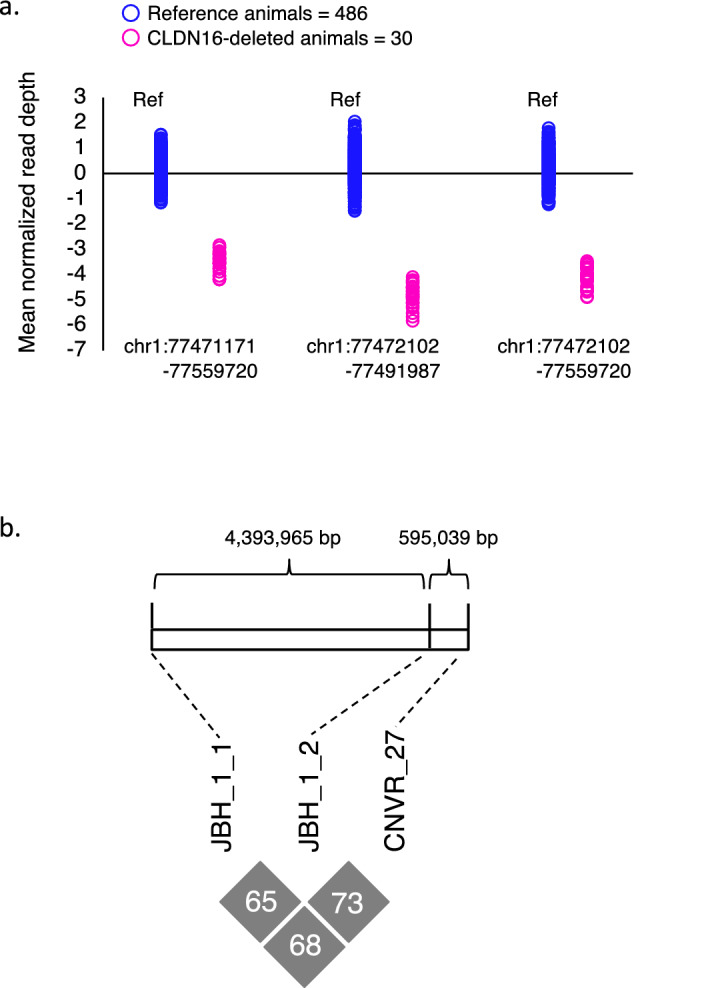
Relationships of CNVR_27 of *CLDN16* in JBH_1_1 and JBH_1_2. (**a**) CNV calling of exome data in three ranges included *CLDN16* exon 1 to exon 4, 77471171–77559720 bp, 77472102–77491987 bp, and 77472102–77559720 bp was performed using XHMM software. Mean normalized read-depth (X-axis) were calculated using the BAM file and plotted. (**b**) CNVR_27 of *CLDN16* was located on the telomeric side at a distance of approximately 4.4 Mb from JBH_1_1 and 0.6 Mb from JBH_1_2. The *r*^2^ values (LD) were estimated by the square of the correlation coefficient (%) in 1980 Japanese Black cattle.

In the seven deleterious recessive haplotypes, no candidates for deleterious SNVs and indels were found in JBH_1_1, JBH_1_2, JBH_4, and JBH_10 based on our criteria described above (Table 4). A previous study in cattle has reported that CNVRs contribute to embryonic mortality^48^, and animals with undesired properties for production are culled^9^. Thus, CNVRs outside the haplotype region may be detected as candidate regions for homozygous haplotype deficiency. We identified 194 CNVRs outside (± 6 Mbp) the risk haplotype regions (see Supplementary Table S8). We detected one CNVR, CNVR_27, in BovineHD BeadChip genotyped data^49^ and exome data (Fig. 4a). CNVR_27 overlapped with a deletion in exon 1 to 5 of *CLDN16*^9^. Cattle with this variant develop severe nephritis and are culled before adulthood. CNVR_27 is located on the telomeric side at a distance of approximately 4.4 Mbp from JBH _1_1 and 0.6 Mbp from JBH_1_2 (Fig. 4b). Additionally, this variant was proven to be in LD with both haplotypes in 1,980 Japanese Black cattle (*r*^2^ = 0.684 and 0.726, respectively) (Fig. 4b). These results suggest that JBH_1_1 and JBH_1_2 can be detected as regions with homozygous haplotype deficiency, although they are located several mega bases outside CNVR_27.

In this study, we did not detect any deleterious mutation associated with pre- and postnatal mortality in JBH_4 and JBH_10 (Table 4). One possibility for this is that gene annotation in these haplotype regions may be flawed in cattle. Recently, RNA sequencing data were accumulated and annotated in RefSeq^50^ and Ensembl^51^ databases; however, the efforts of gene annotation are still underway in cattle, and genes expressed during the pre- and perinatal developmental stages, when mortality frequently occurs, remain largely unknown. Furthermore, we used the exome reagent SureSelect XTBovine all exon, which is designed based on gene annotations reported in 2015; thus, putative causative mutations must be included in the exome capture design for identification using exome-sequencing data. Another possibility is that the reference genome sequence of cattle^52^, which was derived from Hereford cattle, was different from that of Japanese Black cattle, specifically in terms of haplotype regions; thus, exome sequence reads could not be well mapped to the reference genome to call variants. To address these issues, further investigation of the identified haplotype regions using RNA Sequence data of various development stages and de novo assembly of long and short reads of whole genome sequences of carriers in Japanese Black cattle will provide further insights into the causative mutations in this breed.

## Materials and methods

### Ethics

All animal experiments were performed according to the Guidelines for Care and Use of Animals from the University of the Ryukyus, Shimane Prefecture Livestock Technology Center, Shirakawa Institute of Animal Genetics, and Japan Livestock Technology Association. The research protocol was approved by the University of the Ryukyus (#A2018128). Cattle owners provided consent to use the samples and data.

### Genotyping of animals

A total of 4,843 Japanese Black cattle, all over 25 months of age, were genotyped using three BeadChip platforms as follows: 1141 cows, 1,343 steers, and 28 sires were genotyped using Bovine50K BeadChip version 1.0 (Illumina, Cat. #WG-31-120, 54,001 probes); 454 steers were genotyped using Bovine50K BeadChip version 2.0 (Illumina, Cat. #WG-450-2001, 54,609 probes); and 788 cows, 572 steers, and 517 sires were genotyped using BovineHD BeadChip (Illumina, Cat. #WG-450-1002, 777692 probes). The 517 sires genotyped using BovineHD BeadChip were selected for exome sequencing. The animals with a call rate greater than 95% per individual were used in subsequent studies^53^. The SNPs that fulfilled our quality control criteria, which required a call rate greater than 95% per SNP, minor allele frequency (MAF) greater than 0.05, and Hardy–Weinberg equilibrium (HWE) chi-square *P* value greater than 10^–4^, were selected. After applying our criteria, we extracted 32,131 autosomal SNPs that were present on each of the three BeadChip platforms.

### Phasing and haplotype analysis

The genotypes of 32,131 SNPs in 4,843 individuals were phased into diplotypes using BEAGLE 3.3.2^54,55^. Two to 100 SNP windows slides were used across the whole genome to construct haplotypes. The expected number of homozygous individuals for any particular haplotype was calculated using the frequencies of haplotypes and carriers with a binomial test assuming random mating. A *P* value less than 10^–4^ was set as a significance threshold, corresponding to an FDR of 5%.

### Effects of mating type on embryonic mortality rate

Embryonic mortality was determined in cows that received the 2nd AI at 18–29 D, 30–60 D, 61–90 D, 91–140 D, and 141 D–parturition after the 1st AI. The 2nd AI was performed when estrus resumed after the 1st AI in our AI breeding program. The resuming of estrus after the 1st AI indicated that fertilization or conception derived from the 1^st^ AI had failed. Embryonic mortality was estimated based on 785,993 mating records derived from 79,617 reproductive cows^27^. We selected genotyped individuals with SNP arrays. Two types of mating were defined according to the haplotype of the sire and cow: (1, risk mating) mating between a carrier sire and a carrier cow and (2, non-risk mating) mating between other pairs including a non-carrier sire and a non-carrier cow, a carrier sire and a non-carrier cow, and a non-carrier sire and a carrier cow. The segregation ratios from mating were analyzed using a chi-square test.

### Whole exome sequencing

Genomic DNA (200 ng) from 517 sires was used to generate pre-capture libraries, which were prepared using SureSelect XTBovine all exon (Agilent, Cat. #G9496B) and SureSelect XT AUTO reagents (Agilent, Cat. #G9641B) on an Agilent Bravo NGS workstation (Agilent) according to the manufacturer’s instructions. Genomic DNA was sheared using a DNA shearing system (Covaris, Cat. #S220) prior to library preparation. Exome capture probes were designed against 21,664 genes (53.3 Mbp) using the UMD3.1 genome assembly. The sequencing data were generated as 2 × 101 bp reads and processing and base calling were performed using Illumina CASAVA v1.8.2.

Sequence reads (FASTQ files) for each animal were trimmed for adapters, amplified with PCR primers designed using Trimmomatic^56^, and aligned in the UMD3.1 genome assembly^57^ using the Burrows–Wheeler Aligner^58^. BAM files were created using Samtools^59,60^, and duplicate reads were removed using Picard^61^. Average read-depth coverage of the target exome was calculated using BAM files with Picard. Realignment around indels and recalibration were performed using GATK^62^. SNVs and indels were called using Haplotype Caller with GATK. The effects of variants on genes were annotated and predicted using SnpEff^28^ and Ensembl VEP^29^. The possible effect of an amino acid substitution on genes was also predicted using PolyPhen-2^35^ and SIFT^63^, which were included in the VEP analysis.

Accuracy of the 44,772 SNV calls on target exomes of 517 sires, which were loaded on the Illumina Bovine High-Density (HD) BeadChip, was evaluated by comparing the results with SNVs genotyped by arrays using CalcMatch developed by Li^64^.

### Principal component analysis

HD SNP autosomal genotyping data were used for the PCA. The animals with a call rate greater than 95% per individual were used in subsequent studies^53^. The SNPs that fulfilled our quality control criteria, which required a call rate greater than 99%, MAF greater than 0.05, and HWE chi-square *P* value greater than 10^–4^, were selected. After applying our criteria, 593,358 autosomal SNPs were extracted, and a genetic relationship matrix was constructed. The PCA was performed using GCTA^65^.

### Identification of CNVRs

CNVRs were identified based on Illumina BovineHD SNP genotyping data of 1,980 individuals and exome data of 517 Japanese Black cattle sires. CNV calling on HD SNP genotyping data was performed using PennCNV (version June 2011)^34^, which incorporates factors including the log R ratio, B allele frequency, marker distance, and population frequency of the B allele into an HMM. The analysis was performed as described previously^49^. CNV calling on exome data was performed using XHMM software^31,66^, which uses PCA normalization and an HMM to detect and genotype CNVs from normalized read-depth data of target sequences. CNVRs, after merging overlapping with CNVs^32^, were detected using CNVRuler^33^.

### Genotypes of AC_000165.1:g.76542321C>T in *NOL6* and AC_000174.1:g.74743512G>T in *CDC45*

AC_000165.1:g.76542321C>T in *NOL6* was amplified using the following primers: forward primer 5′-AGAGCTGAGGCGGATCATAG-3′ and reverse primer 5′-AGGGGTTTGTGGTCCAGTTT-3′, and the PCR products were digested with *BsrG*I (NEB, Cat. #R0575S). The resulting fragments were separated by electrophoresis on 1.5% agarose gels using 100 bp ladder markers (NEB, Cat. #N3231L). AC_000174.1:g.74743512G > T in *CDC45* was genotyped by direct sequencing of the PCR products using the following primers: forward primer 5′-TGGACAAGCTGTACCACGG-3′ and reverse primer 5′-GAGCACACGAAGGACTTGAG-3′. The PCR products were amplified with using Ex Taq DNA Polymerase (Takara, Cat. #RR006), sequenced using the BigDye Terminator v.3.1 Cycle Sequencing Kit (Applied Biosystems), separated by electrophoresis using the ABI 3730 sequencer (Applied Biosystems), and genotyped using SeqScape V2.5 (Applied Biosystems).

### DNA sequence alignments at the splicing donor and accepter sites in cattle

Data of exon start and end positions in cattle were obtained from GTF files^40^. The sequences of plus and minus strands were extracted considering − 50 bp and + 2 bp from exons as splicing accepters and − 6 bp and + 6 bp from exons as splicing donors. The extracted sequences were matched with the GT/AG mRNA processing rule. A position weight matrix was constructed and plotted using seqLogo^39^.

### Splicing assay using minigene

To assess the effects of AC_000174.1:g.74743512G>T in an intron between exons 14 and 15 (74743369 to 74743910 bp), gDNA was amplified using PrimeSTAR Max DNA Polymerase (Takara, Cat. #R045A), a forward primer (5′-cgGGATCC ggccacaggtctttctcaag-3′; uppercase letters indicate the BamHI linker), and a reverse primer (5′-cgGAATTC tggttgtcccttcctcca-3′; uppercase letters indicate the EcoRI linker). The PCR products were cloned into the BamHI and EcoRI sites of the pcDNA3.1 vector (Invitrogen, Cat. #V79020), which harbors the cytomegalovirus (CMV) promoter. The sequence and orientation of the insert were confirmed by sequencing. The pcDNA3.1 vector was used for mock transfections. Cos-7 cells were cultured in Dulbecco’s modified Eagle’s medium (Sigma, Cat. #D5796) with 10% fetal calf serum (Sigma, Cat. #F-2442) supplemented with 2 mM l-glutamine (Gibco, Cat. #25030-081), 100 units/ml penicillin, and 100 µg/ml streptomycin (Gibco, Cat. #15140-122). Using Lipofectamine 2000 (Invitrogen, Cat. #11668-019), 1 × 10^5^ cells per well were transfected with a mixture of 2 µg of the vector in a 6-well plate. At 48 h after transfection, total RNA was extracted using the RNeasy Mini Kit (Qiagen, Cat. #74104) and treated with DNase I. cDNA was synthesized from 50 ng of RNA using the ReverTra Ace-α Kit (Toyoba, Cat. #FSK-101) with random primers, according to the manufacturer’s instructions. The splicing exons 14 and 15 were detected using the following primers and probe: forward, 5′-CTGCCTCTGCACCAACCT-3′; reverse, 5′-CGAAGGACTTGAGCAGGTGT-3′. PCR products were separated by 2% agarose gel and stained with ethidium bromide, and their fluorescence was detected with an ImageQuant LAS 4000 (GE Healthcare).

### *CDC45* expression analysis

For real-time quantitative PCR, we extracted total RNA from the dermal fibroblasts of homozygotes and heterozygotes of the reference allele using the RNeasy Mini Kit (Qiagen, Cat. #74104) and treated it with DNase I. cDNA was synthesized from 50 ng of RNA using the ReverTra Ace-α Kit (Toyoba, Cat. #FSK-101) with random primers, according to the manufacturer’s instructions. Exon 13 of the *CDC45* gene was detected with the following primers and probe: forward, 5′-AGAGCATAAAGAACAGTTCCGCTA-3′; reverse, 5′-GACTGGCCTGTGAGTGTCAC-3′; and probe, 5′- TCATCCTCATAAACTGTGGCTCGAACGTGG-3′. Exon 20 of the *CDC45* gene was detected with the following primers and probe: forward, 5′-GTTCCTGGATGCGCTCGTG-3′; reverse, 5′-CAACCTAAATAACAGCAAGTCAACAC-3′; and probe, 5′- TGATTTCTTCAGCAGTAACCTCCTCCTCCT-3′. Real-time PCR was performed on a 7900HT Real-Time PCR System (Applied Biosystems) using the comparative Ct method with glyceraldehyde-3-phosphate dehydrogenase mRNA as the internal control.

### Genotypes of embryos from mating between heterozygotes carrying the risk allele (AC_000174.1:g.74743512G>T) of *CDC45*

One sire and four cows heterozygous for AC_000174.1:g.74743512G>T of *CDC45* were selected for mating experiments by a veterinarian at Shimane Prefecture Livestock Technology Center. For estrus synchronization, superovulation, and AI, progesterone was administrated to all cows using a controlled internal drug release (CIDR) device (InterAg, Cat. #Eazi-Breed CIDR-B) between 5 and 10 days from estrus. Estradiol benzoate (1 mg; Kyoritsu Seiyaku Co, Cat. #Estradiol-KS) was injected at 9:00 local time. The day on which the CIDR device was placed in the animal was designated as day 0. At 4 days after the treatment, the cows were serially administered 20 AU of follicle-stimulating hormone (Kyoritsu Seiyaku Co, Cat. #Antorin R∙10) as follows: 5 AU at 9:00 on day 4, 5 AU at 17:00 on day 4, 3 AU at 9:00 on day 5, 3 AU at 17:00 on day 5, 2 AU at 9:00 on day 6, and 2 AU at 17:00 on day 6. On day 6, the PGF2α analog d-cloprostenol (150 µg; Kyoritsu Seiyaku Co, Cat. # Dalmazin) was injected at 16:00, and then the CIDR devices were removed. On day 8, the gonadotropin-releasing hormone analog fertirelin acetate (100 µg; Kyoritsu Seiyaku Co, Cat. #Supolnen) was injected, and the cattle were inseminated with frozen-thawed semen twice, at 16:00 on day 8 and at 9:00 on day 9. At 12 days after AI, ova and embryos were recovered by uterine flushing and observed under a phase-contrast microscope. After determination of the embryonic state and status, each embryo was placed in a 1.5 ml sample tube, centrifuged to remove the supernatant, and frozen at − 80 °C until genotyping. The collected embryos were lysed in 1 × MightyAmp buffer Ver2 (Takara, Cat. #R071A) with 500 µg/ml Proteinase K (Cat. #1.24568.0500) at 55 °C for 3 h and then incubated at 98 °C for 10 min. The lysate (1 µl) was used for the genotyping as described above.

### Screening of the *CDC45* risk allele in Japanese Black cattle

To assess the frequency of the *CDC45* risk allele in Japanese Black cattle in Japan, we genotyped 1,593 steers at 25 to 39 months of age from two central slaughterhouses, which receive animals from across Japan (Tokyo Metropolitan Central Wholesale Market, Tokyo, and Nanko Wholesale Market, Osaka), between 2003 and 2008, as well as 1137 cows from 2.2 to 11.9 years of age from a local subpopulation between 1989 and 2007, in which the carrier sires of the *CDC45* risk allele were originally reared. The AC_000174.1:g.74743512G>T was genotyped by direct sequencing of the PCR products as described above.

## Supplementary Information

Supplementary Information.

## Data Availability

The datasets supporting the results of this article are included within the article and its supplementary files. The data of exome sequencing and SNP array are deposited in the Wagyu Genome Database (WGDB) of the Japan Livestock Technology Association (Yushima, Bunyouku, Tokyo 113-0034, Japan) and managed by the WGDB Consortium. The data in this study are available from WGDB, but restrictions may apply to the availability of these data, which were used under license for the current study. Therefore, the data are not publicly available and may be available from the authors upon reasonable request and with permission from the WGDB.
